# Supplementary material for: Identification of métiers in a multi-gear, multi-species fishery
Source: PLoS One. 2026 Jun 2;21(6):e0348392. doi: 10.1371/journal.pone.0348392 (PMC13229354; doi:10.1371/journal.pone.0348392)

DRB – bivalve dredges

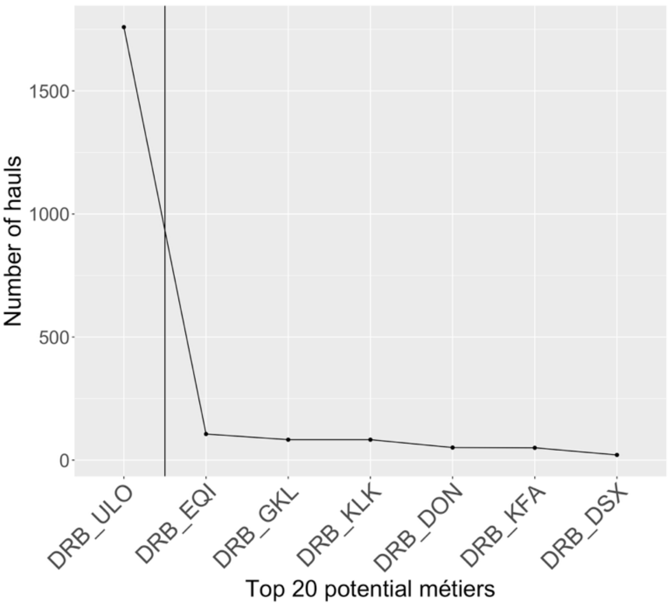

FPO – traps

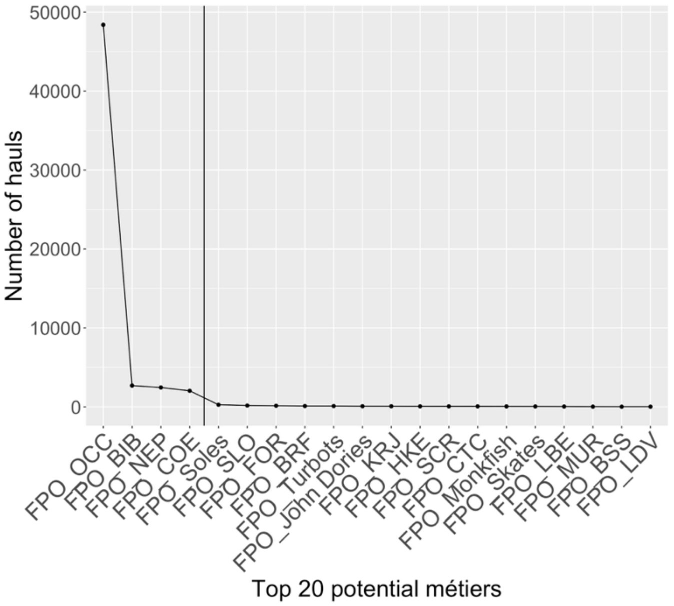

GNS – gillnets

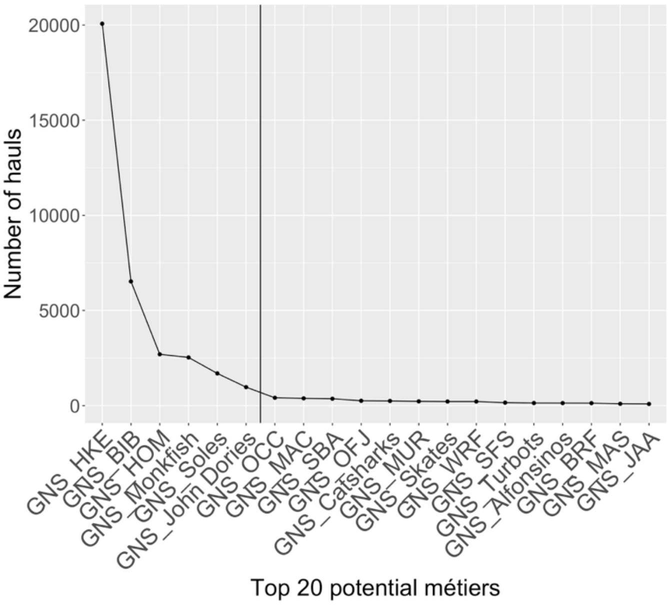

GTR – trammel nets

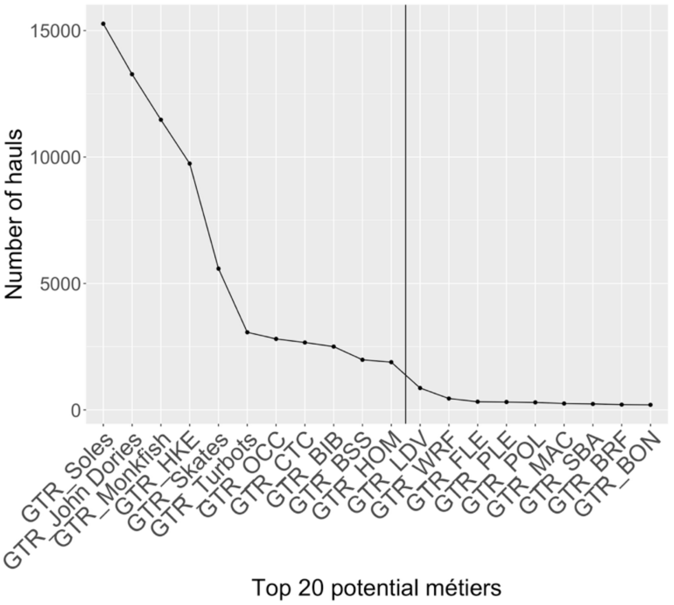

LLD – drifting longline

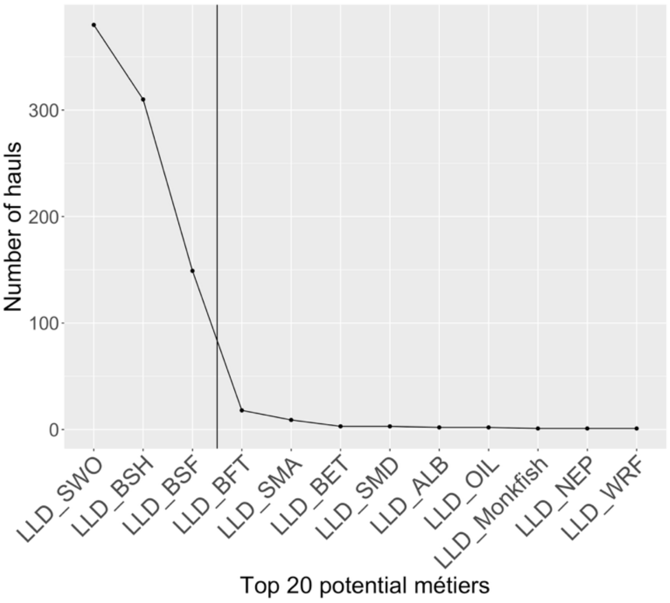

GTR – bottom longline

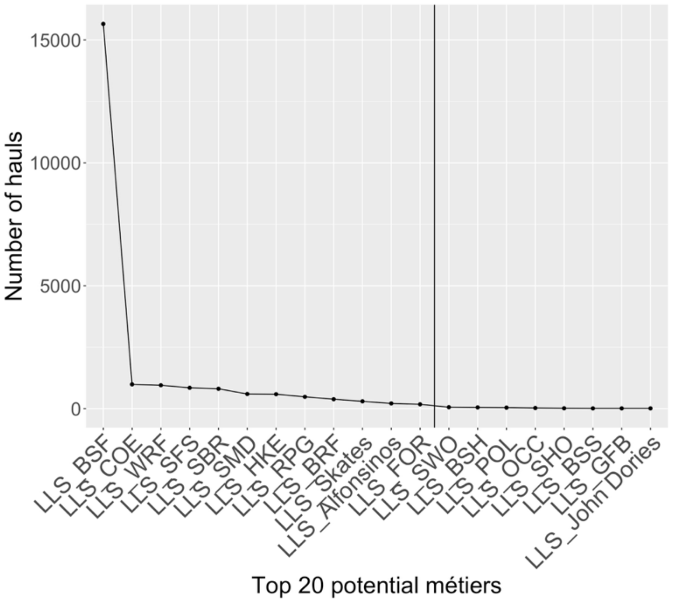

Supplement: S1 Fig — The main gears represented are: DRB – bivalve dredges, FPO – traps, GNS – gillnets, GTR – trammel nets, LLD – drifting longline, and LLS – bottom longline. Only up to 20 métiers of each gear are represented. The vertical dashed line is the cut-off of the point at which the graph curvature stabilized and was used to decide which métiers would be considered for validation. (PDF) [file pone.0348392.s002.pdf]
