# Supplementary material for: Identification of métiers in a multi-gear, multi-species fishery
Source: PLoS One. 2026 Jun 2;21(6):e0348392. doi: 10.1371/journal.pone.0348392 (PMC13229354; doi:10.1371/journal.pone.0348392)
Supplement: Attachment — Submitted filename: PONE-D-25-41485_rev.pdf [file pone.0348392.s006.pdf]

PLOS ONE

Identification of métiers in a multi-gear, multi-species fishery

--Manuscript Draft--

**Manuscript Number:**	PONE-D-25-41485
**Article Type:**	Research Article
**Full Title:**	Identification of métiers in a multi-gear, multi-species fishery
**Short Title:**	Identification of métiers
**Corresponding Author:**	Pedro Leitão, M.D.<br>IPMA: Instituto Portugues do Mar e da Atmosfera<br>Algés, PORTUGAL
**Keywords:**	multi-gear fisheries; métiers identification; electronic logbooks; fishing effort analysis
**Abstract:**	Accurate, gear-specific data are essential for fisheries management to quantify fishing pressure and ecosystem impacts, particularly in multi-gear fleets. This study focuses on the Portuguese multi-gear coastal fleet, which comprises approximately 500 vessels that utilize various types of traps, nets, and longlines to catch around 200 species of fish and invertebrates. The study used electronic logbooks and sales notes from 2014 to 2023 to analyze haul-level information, identifying and validating métiers down to level 6 according to the European Commission's definition (a combination of gear specifications, target species, area of activity, and season). Additionally, regional, seasonal, and temporal trends were associated with each métier. A total of 197,081 hauls were analyzed, resulting in the identification and mapping of 28 métiers across six main gear types. These métiers were validated through previous studies, onboard observations, and interviews with fishers. The study highlights the complexity of quantifying gear-specific fishing effort in multi-gear fisheries, where technical interactions occur and competitive relationships exist between different fishing fleets and gears that exploit the same fish stocks. The importance of digital data collection for robust fisheries management is demonstrated.
**Order of Authors:**	Pedro Leitão
	Margarida Castro
	Aida Campos
**Additional Information:**	
**Question**	**Response**
**Financial Disclosure**<br><br>Enter a financial disclosure statement that describes the sources of funding for the work included in this submission. Review the submission guidelines for detailed requirements. View published research articles from *PLOS ONE* for specific examples.<br><br>This statement is required for submission and **will appear in the published article** if the submission is accepted. Please make sure it is accurate.	Yes

Powered by Editorial Manager® and ProduXion Manager® from Aries Systems Corporation

**Funded studies**<br>Enter a statement with the following details:<br>• Initials of the authors who received each award<br>• Grant numbers awarded to each author<br>• The full name of each funder<br>• URL of each funder website<br>• Did the sponsors or funders play any role in the study design, data collection and analysis, decision to publish, or preparation of the manuscript?<br><br>Did you receive funding for this work?	
Please add funding details.<br>as follow-up to "**Financial Disclosure**<br><br>Enter a financial disclosure statement that describes the sources of funding for the work included in this submission. Review the submission guidelines for detailed requirements. View published research articles from *PLOS ONE* for specific examples.<br><br>This statement is required for submission and **will appear in the published article** if the submission is accepted. Please make sure it is accurate.<br><br>**Funded studies**<br>Enter a statement with the following details:<br>• Initials of the authors who received each award<br>• Grant numbers awarded to each author<br>• The full name of each funder<br>• URL of each funder website<br>• Did the sponsors or funders play any role in the study design, data collection and analysis, decision to publish, or preparation of the manuscript?<br><br>Did you receive funding for this work?"	This study received Portuguese national funds from FCT - Foundation for Science and Technology through projects UIDB/04326/2020 (DOI:10.54499/UIDB/04326/2020), UIDP/04326/2020 (DOI:10.54499/UIDP/04326/2020) and LA/P/0101/2020 (DOI:10.54499/LA/P/0101/2020), P. Leitão was the recipient of a PhD scholarship 2022.11214.BDANA.
Please select the country of your main research funder (please select carefully as in some cases this is used in fee calculation).	PORTUGAL - PT

as follow-up to "**Financial Disclosure**<br><br>Enter a financial disclosure statement that describes the sources of funding for the work included in this submission. Review the submission guidelines for detailed requirements. View published research articles from *PLOS ONE* for specific examples.<br><br>This statement is required for submission and **will appear in the published article** if the submission is accepted. Please make sure it is accurate.<br><br>**Funded studies**<br>Enter a statement with the following details:<br>• Initials of the authors who received each award<br>• Grant numbers awarded to each author<br>• The full name of each funder<br>• URL of each funder website<br>• Did the sponsors or funders play any role in the study design, data collection and analysis, decision to publish, or preparation of the manuscript?<br><br>Did you receive funding for this work?"	
**Competing Interests**<br><br>Use the instructions below to enter a competing interest statement for this submission. On behalf of all authors, disclose any competing interests that could be perceived to bias this work—acknowledging all financial support and any other relevant financial or non-financial competing interests.<br><br>This statement is required for submission and **will appear in the published article** if the submission is accepted. Please make sure it is accurate and that any funding sources listed in your Funding Information later in the submission form are also declared in your Financial Disclosure statement.	NO

View published research articles from *PLOS ONE* for specific examples.<br><br>**NO authors have competing interests**<br><br>Enter: *The authors have declared that no competing interests exist.*<br><br>**Authors with competing interests**<br><br>Enter competing interest details beginning with this statement:<br><br>*I have read the journal's policy and the authors of this manuscript have the following competing interests: [insert competing interests here]*<br><br>* typeset	
**Ethics Statement**<br><br>Enter an ethics statement for this submission. This statement is required if the study involved:<br><br>• Human participants<br>• Human specimens or tissue<br>• Vertebrate animals or cephalopods<br>• Vertebrate embryos or tissues<br>• Field research<br><br>Write "N/A" if the submission does not require an ethics statement.<br><br>General guidance is provided below. Consult the submission guidelines for detailed instructions. **Make sure that all information entered here is included in the Methods section of the manuscript.**	NO

**Format for specific study types**<br><br>**Human Subject Research (involving human participants and/or tissue)**<br>• Give the name of the institutional review board or ethics committee that approved the study<br>• Include the approval number and/or a statement indicating approval of this research<br>• Indicate the form of consent obtained (written/oral) or the reason that consent was not obtained (e.g. the data were analyzed anonymously)<br><br>**Animal Research (involving vertebrate animals, embryos or tissues)**<br>• Provide the name of the Institutional Animal Care and Use Committee (IACUC) or other relevant ethics board that reviewed the study protocol, and indicate whether they approved this research or granted a formal waiver of ethical approval<br>• Include an approval number if one was obtained<br>• If the study involved *non-human primates*, add *additional details* about animal welfare and steps taken to ameliorate suffering<br>• If anesthesia, euthanasia, or any kind of animal sacrifice is part of the study, include briefly which substances and/or methods were applied<br><br>**Field Research**<br><br>Include the following details if this study involves the collection of plant, animal, or other materials from a natural setting:<br>• Field permit number<br>• Name of the institution or relevant body that granted permission	
**Data Availability**<br><br>Authors are required to make all data underlying the findings described fully available, without restriction, and from the time of publication. PLOS allows rare exceptions to address legal and ethical concerns. See the PLOS Data Policy and FAQ for detailed information.	No - some restrictions will apply

A Data Availability Statement describing where the data can be found is required at submission. Your answers to this question constitute the Data Availability Statement and **will be published in the article**, if accepted.<br><br>**Important:** Stating 'data available on request from the author' is not sufficient. If your data are only available upon request, select 'No' for the first question and explain your exceptional situation in the text box.<br><br>Do the authors confirm that all data underlying the findings described in their manuscript are fully available without restriction?	
**Describe where the data may be found in full sentences. If you are copying our sample text, replace any instances of XXX with the appropriate details.**<br><br>• If the data are **held or will be held in a public repository**, include URLs, accession numbers or DOIs. If this information will only be available after acceptance, indicate this by ticking the box below. For example: *All XXX files are available from the XXX database (accession number(s) XXX, XXX.).*<br>• If the data are all contained **within the manuscript and/or Supporting Information files**, enter the following: *All relevant data are within the manuscript and its Supporting Information files.*<br>• If neither of these applies but you are able to provide **details of access elsewhere**, with or without limitations, please do so. For example:<br><br>*Data cannot be shared publicly because of [XXX]. Data are available from the XXX Institutional Data Access / Ethics Committee (contact via XXX) for researchers who meet the criteria for access to confidential data.*<br><br>*The data underlying the results presented in the study are available from (include the name of the third party*	The data is available on request to the Directorate-General for Natural Resources, Safety and Maritime Services

and contact information or URL).<br>• This text is appropriate if the data are owned by a third party and authors do not have permission to share the data.<br><br>* typeset	
Additional data availability information:	

Powered by Editorial Manager® and ProduXion Manager® from Aries Systems Corporation

Manuscript
Click here to access/download;Manuscript;28jun2025 - T2Manuscript.docx

Identification of métiers in a multi-gear, multi-species fishery

Pedro Leitão[1,2*], Margarida Castro[1], Aida Campos[1,2]

[1]Centre of Marine Sciences (CCMAR/CIMAR LA), Campus de Gambelas, Universidade do Algarve, 8005-139 Faro, Portugal
[2]IPMA, Instituto Português do Mar e da Atmosfera, Rua Alfredo Magalhães Ramalho 6, Algés 1495-165, Portugal
*Corresponding author
Email: pedro.leitao@ipma.pt (PL)

Abstract

Accurate, gear-specific data are essential for fisheries management to quantify fishing pressure and ecosystem impacts, particularly in multi-gear fleets. This study focuses on the Portuguese multi-gear coastal fleet, which comprises approximately 500 vessels that utilize various types of traps, nets, and longlines to catch around 200 species of fish and invertebrates. The study used electronic logbooks and sales notes from 2014 to 2023 to analyze haul-level information, identifying and validating métiers down to level 6 according to the European Commission's definition (a combination of gear specifications, target species, area of activity, and season). Additionally, regional, seasonal, and temporal trends were associated with each métier. A total of 197,081 hauls were analyzed, resulting in the identification and mapping of 28 métiers across six main gear types. These métiers were validated through previous studies, onboard observations, and interviews with fishers. The study highlights the complexity of quantifying gear-specific fishing effort in multi-gear fisheries, where technical interactions occur and competitive relationships exist between different fishing fleets and gears that exploit the same fish stocks. The importance of digital data collection for robust fisheries management is demonstrated.

1. Introduction

Fisheries dependent data, such as the type, number, and size of the fishing gear used, as well as the associated catches, are crucial for estimating fishing pressure — a key metric for assessing the environmental impacts of fisheries and informing management advice (Dias et al., 2020; FAO, 1997). In many countries, including those in the European Union, fishers are required to fill logbooks, which can be handwritten or electronic, to record information on fishing operations as part of the fisheries control policy (EU, 2009). This information can estimate fishing effort and determine its spatial distribution (Gerritsen and Lordan, 2011; Tassetti et al., 2022).

In multi-gear fishing fleets, mostly operating fixed gear (traps, nets, and longlines), quantifying gear-specific fishing effort and ecosystem impacts is challenging because most vessels use several types of gears with a wide range of gear characteristics such as net mesh size, hook size, and gear dimensions to catch hundreds of species, making data collection for the multi-gear fleet considerably more complex than for mobile gears such as trawls (González-Álvarez et al., 2016; Monroy et al., 2010; Roditi and Vafidis, 2022). Certain gear types, such as trammel nets and gillnets, are typically low species and size selective (Erzini et al., 2001; Monroy et al., 2010; Roditi and Vafidis, 2022), while others, such as longlines, often target a limited group of species and size ranges, being therefore more selective (Bordalo-Machado and Figueiredo, 2009; Monroy et al., 2010; Roditi and Vafidis, 2022). Furthermore, vessels may use more than one gear type in a single trip, and the same species may be targeted by different gears (Cardoso et al., 2015; Leitão et al., 2025; Szynaka et al., 2022), adding complexity to data collection schemes.

At the European level, characterization of multi-gear fleets using fixed gears is particularly important for southern countries, where the number of active vessels using fixed gears is much higher than in northern countries. Based on the Fleet Register data (EU, 2023a), these numbers are approximately 2,700; 7,000; 3,900; 1,800; and 10,000 for Portugal, Spain, France, Italy, and Greece respectively, compared to the United Kingdom, Ireland, Belgium, Denmark, and

Netherlands, where such numbers are lower (respectively 350; 1,400; <100; 1,400; and 300).

The Portuguese multi-gear fleet, including the local and coastal fleets, comprises over 90% of the total number of vessels in the country, accounts for 36% of the total catch of the main commercially exploited species, and 64% of the total value landed (INE, 2025), making it socially and economically very important. In 2024, the main species landed by this fleet (in weight) were tunas, octopus, black scabbardfish, Pacific chub mackerel, and cuttlefish (INE, 2025). In the coastal segment of the Portuguese multi-gear fleet, which comprises about 500 vessels between 9 and 35 meters in length, about one third are equipped with electronic logbooks (Campos et al., 2021), recording information at the haul level, including the catch (main species and corresponding weights), gear type and location, and timestamp of setting and hauling. This fleet uses a wide variety of fishing gears, differing between geographic regions, ports, and targets different species, often seasonally shifting between gear types (Leitão et al., 2022). For example, black scabbardfish and monkfish are mainly caught in the center and south of Portugal with bottom longlines and gillnets, respectively, and cuttlefish landings by trammel nets are significantly higher in the early months of the year (Leitão et al., 2022).

Previous gear selectivity studies in trammel net fisheries have identified métiers and quantified their respective by-catches (Erzini et al., 2001). However, the lack of a systematic program for collecting data on all important métiers over time limits our understanding of the true scale of the multi-gear fleet's impact on the ecosystem. With the advent of big data access and storage, supervised machine learning algorithms have emerged as valuable tools for identifying fishing patterns and predicting the gear used in multi-gear fisheries (Behivoke et al., 2021; Leitão et al., 2025). Leitão et al. (2025) analyzed information from fishing trips of the Portuguese coastal multi-gear fleet, provided in electronic logbooks and sales notes, along with fleet characteristics. The main fishing gears used were predicted using decision trees with an overall classification error of 14% (Leitão et al., 2025). However, neither the target species nor the technical details of the gear were available for their study, preventing the identification of métiers

down to the level 6 (European Commission, 2010), and consequently their respective fishing effort and environmental impacts.

According to the European Commission Data Collection Framework, a métier is defined as "a group of fishing operations targeting a similar (assemblage of) species, using similar gear, during the same period of the year and/or in the same area, and characterized by a similar exploitation pattern" (European Commission, 2010). The identification of métiers in the multi-gear fleet improves existing knowledge on the dynamics of the fishery, supporting the quantification of the fishing effort as well as its temporal and spatial distribution (Monroy et al., 2010). It also supports the development of management and conservation measures that may include environmental protection (such as spatial and temporal closures for specific gears) without substantially affecting the whole fishing activity.

Several studies have addressed methodological options and respective issues for identifying target species and métiers in the context of multi-gear, multi-species fisheries. A commonly used method is clustering analysis, such as hierarchical k-means clustering, and unsupervised machine learning analysis that requires no prior knowledge of the classification variable used to define the groups. This approach has been applied to sales notes, fishing licenses, and on-board sampling to identify target species or fleet segments (Campos et al., 2021; Duarte et al., 2009; Szynaka et al., 2021; Tzanatos et al., 2006). An intrinsic limitation of this clustering technique is the requirement to predefine the number of groups to a value equal to or less than 30, in the absence of information on the potential number of métiers present (Castro, 2007; Duarte et al., 2009; Kaufman and Rousseeuw, 1990; Okamura et al., 2018), forcing the user to choose a cut-off value based on either high intra-cluster similarity, resulting in many irrelevant métiers, or low intra-cluster similarity, resulting in missing relevant métiers (Tzanatos et al., 2006). On a smaller scale, one study used similarity percentages species contribution analysis (SIMPER), classified trip types, and applied the results to sales notes to identify métiers and characterize fisheries (Monroy et al., 2010). Due to its high computational requirements, this method is rarely used in big data scenarios (Monroy et al., 2010).

The analysis of interviews, combined with administrative data and sales registers from fishing trips, has been used to identify métiers in a small-scale multispecies fishery (González-Álvarez et al., 2016). In this simple and direct approach, the use of métiers over time allowed the identification of new, as well as past métiers at the regional level. Szynaka et al. (2022) used a similar approach and validated 10 métiers of the multi-gear fleet in southern Portugal. However, this approach would be impractical as a long-term, nationwide strategy for obtaining information on gear-specific fishing effort in a multi-gear scenario. This is because it would require intensive sampling, given the differences in seasonality and the large number of ports to be covered.

In the present study, sales notes and electronic logbooks from the period 2012-2023 were used to identify métiers of the multi-gear coastal fleet down to Level 6 (European Commission, 2010). The approach combines the type of gear and its characteristics (such as mesh or hook size) with the target species at the haul level. The target species were assumed to be those that attain a higher sale value. Subsequently, the resulting métiers were validated through previous studies, onboard observations, and interviews with fishers. Lastly, a selection of the most important validated métiers was analyzed for seasonal and regional trends.

2. Material and methods

2.1 Study area

This study focuses on the multi-gear coastal fleet operating in the waters of the Portuguese continental shelf, in southwestern Europe. This area is characterized by the existence of several canyons and strong currents, resulting in a diversity of ecosystems and bottom types (Cardoso et al., 2019). Based on previous studies describing canyons, currents, and prevailing winds (Duarte et al., 2009; Fiúza, 1983; Sousa et al., 2005), the coastline was divided into four geographical areas: 1) northwest, 2) center west, 3) southwest, and 4) south (Fig 1). The northwest and center west regions are separated by the deep Nazaré Canyon, while the center west and southwest regions are divided by the Setubal Canyon off Sesimbra. These different areas are home to more than 200 commercially exploited species (DGRM, 2023).

Fig 1. Map of the Portuguese continental coast. It shows the main landing ports and landmarks, and four distinctive regions: northwest, center west, southwest, and south. Isobaths at 50, 100, 200, 500, 1,000, and 2,000 meters are visible.

2.2 Multi-gear coastal fleet

The Portuguese multi-gear coastal fleet operates in a variety of environments, ranging from the continental shelf to the open sea (Campos et al., 2021). The fleet consists of around 500 vessels measuring over nine meters in length and uses nets, traps, and longlines to catch a wide variety of commercially exploited species. Fishing trips can last from hours to weeks, depending on the vessel’s fishing strategy (Leitão et al., 2025). Most vessels over 12 meters in length fill electronic logbooks that provide information on the fishing gear used and the fishing location of each haul. This haul-level information can be linked to trip sale notes to determine the value of species caught (Leitão et al., 2025). Electronic logbook data, indicating the main gear used, when linked to the species landed, can be used to identify potential métiers and to assess seasonal and geographical patterns of fishing effort.

2.3 Databases used

The data used in the study consisted of electronic logbook records and sales notes from 2014 to 2023 for the coastal multi-gear fleet (the other two segments of the coastal fleet, trawlers and purse seiners, were excluded). When filled in at a haul level, electronic logbooks provide information on individual vessel operations including location (geographical coordinates), timestamps for setting and hauling, main gear used (bivalve towed dredges, traps, gillnets, trammel nets, drifting longlines, and bottom longlines), and main species caught in each haul (by weight). Sales notes (at the trip level) include date, port of landing, and species sold (weight and average price). Average prices from the sales notes enabled the estimation of the contribution of each species to the value of the haul.

Additional information was associated with each haul based on its location (midpoint of setting and hauling), including depth (EMODnet Bathymetry Consortium, 2022) and sediment type (EMODnet Geology, 2022). This information was rasterized to a high level of detail (with a pixel size of less than 0.01 units or 100 meters). Three depth strata were considered: shallow, less than 200 meters; medium, 200 – 500 meters; and deep, over 500 meters. Seven sediment types were used based on the European Marine Observation and Data Network datasets: mud, sandy mud, muddy sand, sand, coarse grain sediment, mixed sediment, and rock and boulder. Hauls with an unknown type of sediment were classified as “unknown”. The depth and sediment type for each haul were assigned using the Sample Raster Values tool, and the resulting file was exported to RStudio.

2.4 Identification of potential métiers

The value contribution of each species was often used to define the target species in previous studies (González-Álvarez et al., 2016; Monroy et al., 2010; Stergiou et al., 2003). Following this approach, the métier associated with each haul was defined as a combination of the gear (including gear details such as mesh and hook size) and the most valuable species caught, which was assumed to be the target species.

The selection of hauls for the multi-gear coastal fleet, used for the identification of métiers, involved the following steps:

1. Selection of hauls in Portuguese waters (FAO subarea 27.9).
2. Exclusion of hauls associated with depths greater than 2,000 meters (outside the area of operation for the fleet of interest); exclusion of hauls associated with trips that did not depart from and return to a port on the Portuguese mainland.
3. Exclusion of hauls with errors or incomplete information, such as reference to miscellaneous fishing gear, zero catches, landing price unavailable, and abnormally high reported weights (i.e., reported weight of hauls sometimes exceeded the maximum sold weight for that species).

The hauls removed in steps 1 to 3 corresponded to 1.8% of the total number, leaving 197,081 hauls. A total of 307 potential métiers were identified (S1 table). Table 1 shows the number of potential métiers for each gear, as well as the number of vessels involved, the number of trips undertaken, and hauls performed.

Table 1. Main gears and respective number of potential métiers

Main gear		Number of potential métiers	Number of vessels	Number of trips	Number of hauls
Gear FAO code	Name				
DRB	Bivalve dredges	7	3	1 062	2153
FPO	Traps	63	103	44 799	57 261
GNS	Gillnets	81	107	28 576	38 508
GTR	Trammel nets	93	111	52 563	75 888
LLD	Drifting longline	12	28	556	879
LLS	Bottom longline	51	64	18 094	22 392
Total		307			197 081

Number of potential métiers by main gear type, and respective number of vessels, trips, and hauls.

The 307 potential métiers were ranked within each gear according to the decreasing number of hauls, and these frequencies were plotted using bar graphs (S1 Fig). For each gear type and based on visual analysis, the point at which the graph curvature stabilized was used to decide which métiers were more important and would be considered for validation.

2.5 Validation of métiers

The most important métiers selected were validated using previous studies, on-board data observations, and interviews with fishers. On-board observations were carried out by trained observers and included interviews with skippers. Such interviews were also conducted in ports and over the telephone. Respondents were asked: "To the best of your knowledge, is species X targeted using fishing gear Y?”. If the answer was yes, the métier was validated. Further characterization of the validated métiers was achieved by adding gear-selective characteristics (e.g., mesh size for nets and traps, or hook number for longlines), as well as associating them with a particular fishing area and/or season. This was based on the location and landing date provided by the electronic logbooks.

2.6 Data analysis

The distribution of fishing effort across the different levels of the variables of interest (year, season, fishing area, depth, and sediment type) was investigated for each validated métier using chi-square goodness-of-fit tests on the haul frequencies. The null hypothesis of uniform fishing effort was tested, and the Bonferroni correction for multiple tests was applied within each variable. Rejection of the null hypothesis was considered for p-values less than 0.01. Data analysis was carried out with the help of RStudio version 2023.09.1+494 and R version 4.4.1 (R Core Team, 2024) with packages "dplyr" (Wickham et al., 2023), "plyr" (Wickham, 2011), "reshape2" (Wickham, 2007), "ggplot2" (Wickham, 2009), "purrr" (Henry and Wickham, 2025), and “stringr” (Wickham, 2023) and QGIS version 3.34.9-Prizren.

3. Results

Out of the 307 potential métiers, the 37 most important ones, based on their significance in terms of haul count, were selected (S1 Fig). The 270 métiers that were excluded had minimal representation with only 6% of the total hauls, averaging 4.5 hauls per métier per year (range from 0.1 to 86.5). Table 2 briefly describes the 37 métiers chosen for validation and characterization: one for bivalve towed dredges, four for traps, six for gillnets, 11 for trammel nets, three for drifting longlines, and 12 for bottom longlines. These 37 métiers are associated with 184,936 hauls.

From the 37 métiers selected for analysis, a total of 28 were validated: 16 through previous studies, nine with interviews with fishers, and three with on-board observations. It was not possible to validate the remaining nine métiers. The distribution of hauls for each of the 28 validated métiers, according to year, season, main fishing area, depth, and sediment type, showed a strong association with these variables. Table 2 presents the most important season, fishing area, and sediment type for each of the identified métiers. S2 Table presents the distribution of the number of hauls by the levels of the following variables: year, season, fishing area, depth, and sediment type. The p-values for the goodness-of-fit test against the uniform distribution of hauls according to year, season, fishing area, depth, and sediment are presented in the S3 Table. For most métiers, the uniform distribution was rejected for all variables, indicating clear differences in fishing intensity concerning these variables (the null hypothesis was rejected in 98% of cases with a p-value < 0.001). However, in two instances — distribution of hauls of bivalve dredges and black scabbardfish bottom longline by season — the null hypothesis failed to be rejected (p-values 0.523 and 0.012, respectively).

The most representative métier of the multi-gear coastal fleet under study is the octopus trap fishery, which is operated year-round, particularly along the northwest coast. This is followed by the hake gillnet fishery in spring and summer, focusing on the NW coast, and the black scabbard fishery, which operates year-round and focuses on the central west coast. Overall, the trammel net fisheries

are the most relevant, targeting soles, John Dories, monkfish, and hake, among others, on a seasonal basis. All these fisheries, except the black scabbard fishery, operate in shallow waters (less than 200 meters) on the continental shelf, in areas with sandy, muddy, or mixed sediments. Longline fisheries, which mostly operate in the central west region at medium or high depths, represent a smaller share.

Table 2. Characterization of the most important métiers down to level 6, with indication of the gear characteristics.

Gear	Name of métier	Number of hauls	Mesh size/hook number	Main season	Main fishing area	Main depth	Main sediment	Validation through
DRB	Solid surf clam bivalve dredges	1 759	-	All seasons	NW	S	Sand	(Rufino et al., 2008)
FPO	Common octopus traps	48 390	8 – 50 or none*	-	NW	S	-	(Szynaka et al., 2022)
FPO	Pouting traps	2 697	50	-	NW	S	Mixed sediment	Interviews
FPO	Norway lobster traps	2 461	30 – 50	-	CW	M	Muddy sand	(Castro et al., 2003)
FPO	European conger traps	2 038	50	-	NW	S	-	Interviews
GNS	European hake gillnets	20 069	100	-	NW	S	-	(Santos et al., 2003)
GNS	Pouting gillnets	6 526	60	-	NW	S	Mixed sediment	(Figueiredo et al., 2020)
GNS	Atlantic horse mackerel gillnets	2 698	-	-	-	-	-	Not validated
GNS	Monkfish gillnets	2 533	220	-	-	S	Muddy sand	(Fonseca et al., 2005; Szynaka et al., 2022)
GNS	Soles gillnets	1 692	60	-	-	S	-	(Szynaka et al., 2022)
GNS	John Dories gillnets	972	200	-	NW	S	-	Interviews
GTR	Soles trammel nets	15 269	80-100	-	NW	S	-	(Karim Erzini et al., 2001)
GTR	John Dories trammel nets	13 273	200	-	-	S	-	On-board observations
GTR	Monkfish trammel nets	11 474	220	-	-	S	Muddy sand	(Karim Erzini et al., 2001)
GTR	European hake trammel nets	9 740	-	-	-	-	-	Not validated
GTR	Skates trammel nets	5 586	220	-	NW	S	-	(Figueiredo et al., 2020)

GTR	Turbots trammel nets	3 070	200	-	NW	S	-	On-board observations
GTR	Common octopus trammel nets	2 808	-	-	-	-	-	Not validated
GTR	Common cuttlefish trammel nets	2 665	80-100	Winter	NW	S	-	(Karim Erzini et al., 2001; Szynaka et al., 2022)
GTR	Pouting trammel nets	2 503	-	-	NW	-	-	Not validated
GTR	European seabass trammel nets	1 982	80	Winter	-	S	-	Interviews
GTR	Atlantic horse mackerel trammel nets	1 888	-	-	-	-	-	Not validated
LLD	Swordfish drifting longline	380	16/0 and 17/0	Autumn	CW	D	Unknown	(Parra et al., 2023)
LLD	Blue shark drifting longlines	310	16/0 and 17/0	Summer	NW	S	-	(Parra et al., 2023)
LLD	Black scabbardfish drifting longlines	149	-	-	-	-	-	Not validated
LLS	Black scabbardfish bottom longlines	15 652	5 to 8	All seasons	CW	D	Unknown	(Bordalo-Machado and Figueiredo, 2009)
LLS	European conger bottom longlines	990	3 to 8	-	-	S	-	Interviews
LLS	Wreckfish bottom longlines	954	6 to 7	-	CW	-	-	(Campos et al., 2019)
LLS	Silver scabbardfish bottom longlines	849	9	-	CW	M	Rock and boulders	Interviews
LLS	Blackspot seabream bottom longlines	810	8 to 9	-	CW	M	-	On-board observations

LLS	Smooth-hound bottom longlines	598	-	-	-	-	-	Not validated
LLS	European hake bottom longlines	590	7 to 9	-	CW	M	-	(K Erzini et al., 2001)
LLS	Red porgy bottom longlines	482	8 to 9	-	CW	S	Rock and boulders	Interviews
LLS	Blackbelly rosefish bottom longlines	387	3 to 5	-	-	-	-	Interviews
LLS	Skates bottom longlines	300	-	-	-	-	-	Not validated
LLS	Alfonsinos bottom longlines	214	6	-	CW	M	-	Interviews
LLS	Forkbeard bottom longlines	178	-	-	-	-	-	Not validated

Main fishing areas: CW - center west, NW - northwest, SW - southwest, and S - south. **Depth**: S - shallow, less than 200 meters, M - medium, 200–500 meters, D - deep, more than 500 meters. Unvalidated métiers are highlighted in grey.

Most métiers showed increased activity at certain times of the year. However, some were found to be strongly seasonal, with over 50% of the hauls occurring in a single season. Examples of the latter include the cuttlefish and European seabass trammel net fisheries, which operate in winter, and the swordfish and blue shark drifting longline fisheries, which operate in autumn and summer, respectively.

Certain métiers are strongly associated with specific depths. Most trap, gillnet, and trammel net fisheries operate almost exclusively in shallow waters. Red porgy and European conger bottom longlines are also used in shallow waters. Silver scabbardfish, blackspot seabream, European hake, and alfonsinos are caught with bottom longlines at depths of up to 500 meters. Swordfish drifting longlines, and black scabbardfish bottom longlines in deep waters.

Métiers were also found to be associated with sediment type. Traps were often used in mixed sediments, whereas drifting or bottom longlines were used in muddy sand, rocks, and boulders. The métiers most strongly associated with certain sediments are pouting traps and gillnets in mixed sediments, monkfish trammel nets and gillnets in muddy sand, and bottom longlines for silver scabbardfish and red porgy in rocky areas (S2 Table).

Lastly, yearly fluctuations were observed in most métiers during the study period, including those involving traps for octopus, pouting, and conger, as well as most gillnet, trammel net, and bottom longline métiers (S2 Table). An increase in fishing activity was observed for turbot with trammel nets, wreckfish and silver scabbardfish with bottom longlines, and for swordfish with drifting longlines. Fishing activity for black scabbardfish was found to be constant. Conversely, trammel net fishing decreased for monkfish and skates.

Figure 2 shows heat maps of the fishing pressure on selected target species (pouting, European conger, European hake, monkfish, and John Dories) that were targeted by more than one type of fishing gear. There is a clear association between a group of métiers and the area fished. Gillnet and bottom longline fisheries for hake are prevalent in the northwest and the central west,

respectively. Hake gillnets are primarily used between Nazaré and Peniche, with high fishing pressure in the Nazaré Canyon, but expanding to Sagres, in the south, and Viana do Castelo, in the north; the longline fishery for hake operates far from the coast, in deep waters to the west of Peniche. In contrast, bottom gillnets for monkfish are used in the southwest and south areas, between Sesimbra and Vila Real de Sto António, while trammel nets for monkfish and John Dories operate mainly in the center west and southwest, between Peniche and Sagres. Trap and gillnet fisheries for pouting, and bottom longlines for European conger are exclusively found in the northwest.

Fig 2. Heat maps of the fishing pressure of 10 identified métiers. Selected target species were pouting, European conger, European hake, monkfish, and John Dories. The data combines information from 2014 to 2023.

4. Discussion

In this study, a total of 28 métiers were identified and validated, many of which for the first time (to the best of our knowledge) at Level 6 (European Commission, 2010). This is particularly important in a multi-gear fleet such as the one studied here, where technical interactions occur and competitive relationships exist between different fishing fleets and gears exploiting the same stocks. These interactions can be complex, involving direct competition for the same species, as well as the capture of non-target species. This applies to the European hake, pouting, and monkfish, which can be caught using the same gear, but with different selectivity characteristics, resulting in varying levels of by-catch. Separating the fishing effort for different gears targeting the same species is crucial for effective fisheries management, since the actions of one fleet impact the catch and profitability of others.

The distribution of hauls for each of the 28 validated métiers showed a strong association with variables such as year, season, main fishing area, depth, and sediment type. Traps and nets were mostly used in the northwest area, in mixed sediments or muddy sand, whereas longlines mostly operated in the central West area, in steeper slopes or rocky areas. During the interviews, fishers consistently reported avoiding using nets and traps in these areas due to the high risk of their getting stuck or entangled. One difference between the trap/net and longline métiers is that the former is more season-specific, usually restricted to summer or winter. In contrast, longline fisheries are spread more evenly across seasons, while not necessarily consecutive. We hypothesize that these differences may be related to variations in species availability in distant and close-to-shore waters. Traps and gillnets are primarily used in shallow waters close to the shore. The main target species for these métiers, such as hake, soles, and cuttlefish, are known to migrate between coastal and deeper waters for reproduction (Batista et al., 2009; K Erzini et al., 2001). Consequently, the availability of fish varies by season depending on the fishing methods used. This is the case for hake, which is targeted with longlines in the spring and with gillnets and trammel nets in the summer.

Interannual fluctuations in the number of hauls were observed in most métiers; however, some gained importance, while others declined. The trammel nets fishery towards turbots, mainly targeting turbot (*Scophthalmus maximus*) and brill (*Scophthalmus rhombus*), steadily increased from 50 hauls in 2014 to 550 in 2023. This is in line with the turbots landings data for the same period, which increased by approximately 300% in weight and 400% in value (INE, 2025). The growing interest in this fishery could result from both turbot species being among the top ten species landed in Portugal with higher value per kilogram (INE, 2025), making it increasingly more popular among fishers. We found that the turbots fishery is mostly associated with seven vessels that alternate their activity between octopus traps and soles trammel nets, indicating that turbots fishing was added to the list of métiers they use.

High interannual variability in the number of hauls using traps and gillnets for pouting through the time series might indicate them as being last resort métiers directed at a highly fluctuating species. Pouting (*Trisopterus luscus*) is a short-lived species, usually reaching 3 to 4 years of age, with higher abundance in northern Europe and some areas of the Mediterranean (Cohen et al., 1990). In 2019, stock assessment for this species lacked sufficient data but showed an increasing fishing pressure and a decreasing trend in biomass (Moreno et al., 2020). Due to the high variability in catches and their commercial importance, especially in the northwest part of Portugal, further studies on stock assessment and fishing effort are warranted.

A detailed analysis of the temporal and regional distribution of fishing effort was conducted, excluding activities involving bivalve dredges, drifting longlines for swordfish, and bottom longlines for black scabbardfish. The reason is that these activities had already been well characterized in previous studies (Campos et al., 2023; Gaspar, 2017; Leitão et al., 2021; Rufino et al., 2023; Santos et al., 2013). The blue shark (*Prionace glauca*) is often a bycatch of the drifting longline fishery but has recently been identified as a target species (Parra et al., 2023). Our results agree with recent findings by identifying hauls targeting blue shark with distinct seasonality, geographical area, and depth from those targeting swordfish.

Of the nine métiers that were not validated, we propose that seven resulted from misidentification the target species. The target species was the one associated with the highest-value catches within a given haul. In some cases, a common bycatch species may have achieved the highest commercial value and been mistakenly identified as the target species. This was the case of Atlantic horse mackerel (*Trachurus trachurus*) bycatch, frequently present in European hake and pouting gillnets (Fonseca et al., 2005), and common octopus (*Octopus vulgaris*) bycatch in soles and cuttlefish trammel nets (Batista et al., 2009). Similarly, forkbeards and skates are among the top ten most economically important species/groups on bottom longlines. Given their economic importance, at the initial stage of our procedure, they were identified as potential target species. This underlines the importance of the validation process of métiers.

For the remaining two unvalidated métiers - drifting longlines for black scabbardfish (*Aphanopus carbo*) and bottom longlines for smooth hound (*Mustelus mustelus*) - we assumed that the gear registered in the electronic logbook was incorrect. We compared the entries in the electronic logbooks for drifting longlines and bottom longlines for black scabbardfish. The drifting longline hauls for black scabbardfish come from one vessel in 2019; this vessel used bottom longlines for black scabbardfish in all other years. The species composition, catches, and fishing areas are identical to those of bottom longlines targeting black scabbardfish on all other vessels. Although this was not confirmed with the skipper of the relevant vessel, we hypothesize that it most likely resulted from a systematic error in the gear selected when filling in the electronic logbooks. Similarly, the bottom longline fishery for smooth hound could not be validated. According to our data, the last fishing hauls were in 2020, at depths of around 1500 meters, which is inconsistent with the biology of smooth hound, as it is a demersal shark that inhabits depths of around 50 meters and up to 600 meters.

Regarding the trammel nets fishery targeting soles and cuttlefish, although it has been studied before (Erzini et al., 2001; Szynaka et al., 2022), further research is needed to determine whether it represents a single or distinct métiers. Both species are targeted with trammel nets of 80 to 100 mm mesh size, in sandy bottoms, mainly in the northwest region. Batista et al. (2009) studied the trammel

net fishery for soles and cuttlefish and showed that while soles are available to the fishery all year round, cuttlefish availability is lower, between spring and summer, possibly due to their inshore-offshore migrations. The methodology used here may have led to the identification of one métier where the cuttlefish is less abundant (identifying soles as the target species) and another one when the cuttlefish is more abundant (identifying cuttlefish as the target species).

A total of ten species of skates are landed in mainland Portuguese fishing ports (Figueiredo et al., 2020). In this study, all these species were included in a single group, the family Rajidae. This is because it has been demonstrated that most ray species are misidentified during landings (Serra-Pereira, 2010). The most important species of skates in terms of weight landed are the blonde skate (*Raja brachyura*) at 52%, the thornback skate (*Raja clavata*) at 22%, and the spotted skate (*Raja montagui*) and undulate skate (*Raja undulata*) both at 7% (Serra-Pereira, 2010). According to IUCN[1], three of these species (*R. brachyura, R. clavata, and R. undulata*) are Near Threatened, while *R. montagui* is of Least Concern. Of the remaining six species identified in Figueiredo et al. (2020), two are Least Concern (*Leucoraja naevus* and *Raja miraletus*), one is Near Threatened (*Raja microocellata*), one is Vulnerable (*Dipturus oxyrinchus*), and two are Endangered (*Dipturus oxyrinchus* and *Leucoraja circularis*). Since 2017, the national sampling program (PNAB) has fixed the misidentification of skates in the center and northern ports (ICES, 2024); nevertheless, efforts should be made to improve the identification by landing personnel in national fishing ports, ultimately to implement conservation measures for the most vulnerable species.

This study was based on approximately 150 vessels, accounting for about one-third of the multi-gear coastal fleet. Information on the remaining fleet is unavailable because for smaller vessels, the DGRM only requires handwritten logbooks, which were not analyzed here. Previous work by Leitão et al. (2025) compared the catch compositions, for the same main gear, in coastal vessels with and without electronic logbooks and found no significant differences between

[1] https://www.iucnredlist.org accessed 2025-07-16. Status defined for global or Atlantic distribution of species.

the two groups. Therefore, we are confident that the fleet analyzed in this study is a representative sample of the entire coastal fleet. However, this representation does not necessarily extend to the local fleet, whose vessels are generally smaller than nine meters in length.

Analyzing electronic logbooks is crucial for characterizing and estimating fishing effort, as well as for managing the EU's multi-gear fleets. The detailed information about métiers down to level 6, their gear characteristics, and temporal and geographic fishing effort distribution associated with each vessel provides valuable data for improving the efficiency of sampling programs. In fact, with few exceptions, métiers cannot be assigned individual vessels but are rather a reflection of the activity undergone by each vessel at a specific time in a certain area. The common situation is to have groups of vessels switching between métiers, as they target different components of the fish assemblages at different times.

Fishery-based information, as it has been used within the scope of ICES, has not considered the existence of these directed fisheries; their integration as stratification levels in sampling programs is crucial to obtaining more accurate species-specific indices of abundance, fishing effort, and discards, which can be used in stock assessment. Moreover, applying this knowledge to fisheries management improves the balance between the exploitation and conservation of marine resources in coastal areas, especially when complemented by the ecological footprint of each gear (e.g., bycatch and capture of protected species). At the regional level, management can entail closing areas and seasons to specific gears while still allowing fishing activity in other areas and periods. This approach provides information to protect biodiversity, conserve exploited resources, and maintain the economic viability of multi-gear coastal fleets. The implementation of electronic logbooks and monitoring devices in all fishing vessels required by the EU by 2030 (EU, 2023b) will improve the quality of the information necessary for this.

Acknowledgments

This study received Portuguese national funds from FCT - Foundation for Science and Technology through projects UIDB/04326/2020 (DOI:10.54499/UIDB/04326/2020), UIDP/04326/2020 (DOI:10.54499/UIDP/04326/2020) and LA/P/0101/2020 (DOI:10.54499/LA/P/0101/2020), P. Leitão was the recipient of a PhD scholarship 2022.11214.BDANA.

The bathymetric metadata and Digital Terrain Model data products have been derived from the EMODnet Bathymetry portal - http://www.emodnet-bathymetry.eu. The authors thank the Directorate-General for Natural Resources, Safety and Maritime Services, and the fishers interviewed in this work for the data provided.

References

Batista, M.I., Teixeira, C.M., Cabral, H.N., 2009. Catches of target species and bycatches of an artisanal fishery: The case study of a trammel net fishery in the Portuguese coast. Fish Res 100, 167–177. https://doi.org/10.1016/j.fishres.2009.07.007

Behivoke, F., Etienne, M.-P., Guitton, J., Randriatsara, R.M., Ranaivoson, E., Léopold, M., 2021. Estimating fishing effort in small-scale fisheries using GPS tracking data and random forests. Ecol Indic 123, 107321. https://doi.org/10.1016/j.ecolind.2020.107321

Bordalo-Machado, P., Figueiredo, I., 2009. The fishery for black scabbardfish (Aphanopus carbo Lowe, 1839) in the Portuguese continental slope. Rev Fish Biol Fish 19, 49–67. https://doi.org/10.1007/s11160-008-9089-7

Campos, A., Fonseca, P., Araújo, G., Parente, J., 2021. Definition of landing profiles in the Portuguese coastal multi-gear fleet, in: Developments in Maritime Technology and Engineering. CRC Press, London, pp. 693–697. https://doi.org/10.1201/9781003216599-73

Campos, A., Leitão, P., Sousa, L., Gaspar, P., Henriques, V., 2023. Spatial patterns of fishing activity inside the Gorringe bank MPA based on VMS, AIS and e-logbooks data. Mar Policy 147, 105356. https://doi.org/10.1016/j.marpol.2022.105356

Campos, A., Lopes, P., Fonseca, P., Figueiredo, I., Henriques, V., Gouveia, N., Delgado, J., Gouveia, L., Amorim, A., Araujo, G., Drago, T., dos Santos, A., 2019. Portuguese fisheries in seamounts of Madeira-Tore (NE Atlantic). Mar Policy 99, 50–57. https://doi.org/10.1016/j.marpol.2018.10.005

Cardoso, I., Moura, T., Mendes, H., Silva, C., Azevedo, M., 2015. An ecosystem approach to mixed fisheries: technical and biological interactions in the Portuguese multi-gear fleet. ICES Journal of Marine Science 72, 2618–2626. https://doi.org/10.1093/icesjms/fsv138

Cardoso, P.G., Dolbeth, M., Sousa, R., Relvas, P., Santos, R., Silva, A., Quintino, V., 2019. The Portuguese Coast, in: World Seas: An Environmental Evaluation. Elsevier, pp. 189–208. https://doi.org/10.1016/B978-0-12-805068-2.00009-7

Castro, J., 2007. Identification and Segmentation of Mixed-Species Fisheries Operating in the Atlantic Iberian Peninsula Waters. European Commision - Directorate General for Fisheries and maritime Affairs 1–260.

Castro, M., Araújo, A., Monteiro, P., Madeira, A.M., Silvert, W., 2003. The efficacy of releasing caught Nephrops as a management measure, in: Fisheries Research. Elsevier, pp. 475–484. https://doi.org/10.1016/j.fishres.2003.09.033

Cohen, D.M., Inada, T., Iwamoto, T., Scialabba, N., 1990. Gadiform fishes of the world (Order Gadiformes). An annotated and illustrated catalogue of cods, hakes, grenadiers and other gadiform fishes known to date., FAO species catalogue.

DGRM, 2023. Multi-gear fisheries data from 2012 to 2023. Daily sales, electronic logbooks, vessels licenses and characteristics delivered on request [dataset].

Dias, V., Oliveira, F., Boavida, J., Serrão, E.A., Gonçalves, J.M.S., Coelho, M.A.G., 2020. High Coral Bycatch in Bottom-Set Gillnet Coastal Fisheries Reveals Rich Coral Habitats in Southern Portugal. Front Mar Sci 7, 1–16. https://doi.org/10.3389/fmars.2020.603438

Duarte, R., Azevedo, M., Afonso-Dias, M., 2009. Segmentation and fishery characteristics of the mixed-species multi-gear Portuguese fleet. ICES Journal of Marine Science 66, 594–606. https://doi.org/10.1093/icesjms/fsp019

EMODnet Bathymetry Consortium, 2022. EMODnet Digital Bathymetry. EMODnet Bathymetry Consortium. https://doi.org/10.12770/ff3aff8a-cff1-44a3-a2c8-1910bf109f85

EMODnet Geology, 2022. EMODnet Seabed Substrates. EMODnet Geology.

Erzini, K, Gonçalves, J.M.S., Bentes, L., Lino, P.G., Ribeiro, J., 2001. The hake deepwater semi-pelagic (“pedra-bola”) longline fishery in the Algarve (southern Portugal). Fish Res 51, 327–336. https://doi.org/10.1016/S0165-7836(01)00256-9

Erzini, Karim, Stergiou, K.I., Puente, E., Hernando, J.A., 2001. Trammel net selectivity studies in the Algarve (southern Portugal), Gulf of Cadiz (Spain), Basque Country (Spain) and Cyclades Islands (Greece).

EU, 2023a. Fleet Register [WWW Document]. URL https://webgate.ec.europa.eu/fleet-europa/search_en

EU, 2023b. Regulation (EU) 2023/2842 of the European Parliament and of the Council of 22 November 2023 amending Council Regulation (EC) No 1224/2009, and amending Council Regulations (EC) No 1967/2006 and (EC) No 1005/2008 and Regulations (EU) 2016/1139, (EU) 2017/2 2842, 1–105.

EU, 2009. Council Regulation (EC) No 1224/2009. Establishing a Community control system for ensuring compliance with the rules of the common fisheries policy. Official Journal of the European Union L343, 1–50.

European Commission, 2010. Commission Decision 2010/93/EU.

FAO, 1997. Fisheries management, FAO Techinal Guidelines For Resposible Fisheries.

Figueiredo, I., Maia, C., Lagarto, N., Serra-Pereira, B., 2020. Bycatch estimation of Rajiformes in multispecies and multigear fisheries. Fish Res 232, 105727. https://doi.org/10.1016/j.fishres.2020.105727

Fiúza, A.F.G., 1983. Upwelling Patterns off Portugal, in: Coastal Upwelling Its Sediment Record. Springer US, Boston, MA, pp. 85–98. https://doi.org/10.1007/978-1-4615-6651-9_5

Fonseca, P., Martins, R., Campos, A., Sobral, P., 2005. Gill-net selectivity off the Portuguese western coast. Fish Res 73, 323–339. https://doi.org/10.1016/j.fishres.2005.01.015

Gaspar, M., 2017. Razor clams fisheries (Ensis siliqua y Solen marginatus) in Portugal.

Gerritsen, H., Lordan, C., 2011. Integrating vessel monitoring systems (VMS) data with daily catch data from logbooks to explore the spatial distribution of catch and effort at high resolution. ICES Journal of Marine Science 68, 245–252. https://doi.org/10.1093/icesjms/fsq137

González-Álvarez, J., García-de-la-Fuente, L., García-Flórez, L., Fernández-Rueda, M. del P., Luis Alcázar-Álvarez, J., 2016. Identification and Characterization of Métiers in Multi-Species Artisanal Fisheries. A Case Study in Northwest Spain. Natural Resources 07, 295–314. https://doi.org/10.4236/nr.2016.76026

Henry, L., Wickham, H., 2025. Purrr: Functional programming tools. R package version 1.0.4.

ICES, 2024. Report of the Working Group on Elasmobranch Fishes (WGEF). ICES Scientific Reports. https://doi.org/10.17895/ices.pub.26935504

INE, 2025. Estatísticas da Pesca - 2024.

Kaufman, L., Rousseeuw, P., 1990. Clustering Large Applications (Program CLARA), in: Finding Groups in Data: An Introduction to Cluster Analysis. John Wiley & Sons, Inc., New Jersey, pp. 126–163. https://doi.org/10.1002/9780470316801.ch3

Leitão, P., Campos, A., Castro, M., 2025. Predicting gear used in a multi-gear coastal fleet. Fish Res 281, 107199. https://doi.org/10.1016/j.fishres.2024.107199

Leitão, P., Sousa, L., Castro, M., Campos, A., 2022. Time and spatial trends in landing per unit of effort as support to fisheries management in a multi-gear coastal fishery. PLoS One 17, e0258630. https://doi.org/10.1371/journal.pone.0258630

Leitao, P., Sousa, L., Henriques, V., Gaspar, P., Campos, A., 2021. Fishing activities in the Gorringe bank, a Portuguese Marine Protected Area. World Fisheries Congress 2021 15.

Monroy, C., Salas, S., Bello-Pineda, J., 2010. Dynamics of Fishing Gear and Spatial Allocation of Fishing Effort in a Multispecies Fleet. N Am J Fish Manag 30, 1187–1202. https://doi.org/10.1577/m09-101.1

Moreno, A., Rocha, A., Silva, A., Melo, A.A., Pereira, B., Silva, C., Feijó, D., Soares, E., Borges, F., Mendes, H., Farias, I., Figueiredo, I., Wise, L., Azevedo, M., Gaspar, M.B., Gonçalves, P., Lino, P., Alpoim, R., Coelho, R., Garrido, S., Moura, T., Stratoudakis, Y., 2020. Estado dos stocks em 2019 e aconselhamento científico para a sua gestão em 2020. Lisbon.

Okamura, H., Morita, S.H., Funamoto, T., Ichinokawa, M., Eguchi, S., 2018. Target-based catch-per-unit-effort standardization in multispecies fisheries. Canadian Journal of Fisheries and Aquatic Sciences 75, 452–463. https://doi.org/10.1139/cjfas-2016-0460

Parra, H., Pham, C.K., Machete, M., Santos, M., Bjorndal, K.A., Vandeperre, F., 2023. The Portuguese industrial pelagic longline fishery in the Northeast Atlantic: Catch composition, spatio-temporal dynamics of fishing effort, and target species catch rates. Fish Res 264. https://doi.org/10.1016/j.fishres.2023.106730

R Core Team, 2024. R: A language and environment for statistical computing.
Roditi, K., Vafidis, D., 2022. Small-scale fisheries in the south Aegean Sea: métiers and associated economics. Ocean Coast Manag 224, 106185. https://doi.org/10.1016/j.ocecoaman.2022.106185
Rufino, M.M., Gaspar, M.B., Maynou, F., Monteiro, C.C., 2008. Regional and temporal changes in bivalve diversity off the south coast of Portugal. Estuar Coast Shelf Sci 80, 517–528. https://doi.org/10.1016/j.ecss.2008.09.014
Rufino, M.M., Mendo, T., Samarão, J., Gaspar, M.B., 2023. Estimating fishing effort in small-scale fisheries using high-resolution spatio-temporal tracking data (an implementation framework illustrated with case studies from Portugal). Ecol Indic 154, 110628. https://doi.org/10.1016/j.ecolind.2023.110628
Santos, M.N. Dos, Gaspar, M., Monteiro, C.C., Erzini, K., 2003. Gill net selectivity for European hake Merluccius merluccius from southern Portugal: implications for fishery management. Fisheries Science 69, 873–882. https://doi.org/10.1046/j.1444-2906.2003.00702.x
Santos, M.N., Coelho, R., Lino, P.G., 2013. Standardized CPUE for swordfish (xiphias gladius) caught by the portuguese pelagic longline fishery in the north Atlantic 70, 1783–1791.
Serra-Pereira, B., 2010. Skates and rays diversity, exploration and conservation - case-study of the thornback ray, Raja clavata.
Sousa, P., Azevedo, M., Gomes, M.C., 2005. Demersal assemblages off Portugal: Mapping, seasonal, and temporal patterns. Fish Res 75, 120–137. https://doi.org/10.1016/j.fishres.2005.03.012
Stergiou, K.I., Machias, A., Somarakis, S., Kapantagakis, A., 2003. Can we define target species in Mediterranean trawl fisheries? Fish Res 59, 431–435. https://doi.org/10.1016/S0165-7836(02)00016-4
Szynaka, M.J., Erzini, K., Gonçalves, J.M.S., Campos, A., 2021. Identifying Métiers Using Landings Profiles: An Octopus-Driven Multi-Gear Coastal Fleet. J Mar Sci Eng 9, 1022. https://doi.org/10.3390/jmse9091022
Szynaka, M.J., Fernandes, M., Anjos, M., Erzini, K., Gonçalves, J.M.S., Campos, A., 2022. Fishers, Let Us Talk: Validating Métiers in a Multi-Gear Coastal Fishing Fleet. Fishes 7. https://doi.org/10.3390/fishes7040174

Tassetti, A.N., Galdelli, A., Pulcinella, J., Mancini, A., Bolognini, L., 2022. Addressing Gaps in Small-Scale Fisheries: A Low-Cost Tracking System. Sensors 22, 839. https://doi.org/10.3390/s22030839

Tzanatos, E., Somarakis, S., Tserpes, G., Koutsikopoulos, C., 2006. Identifying and classifying small-scale fisheries métiers in the Mediterranean: A case study in the Patraikos Gulf, Greece. Fish Res 81, 158–168. https://doi.org/10.1016/j.fishres.2006.07.007

Wickham, H., 2023. stringr: Simple, Consistent Wrappers for Common String Operations. CRAN: Contributed Packages. https://doi.org/10.32614/CRAN.package.stringr

Wickham, H., 2011. The Split-Apply-Combine Strategy for Data Analysis. J Stat Softw 40, 1–29. https://doi.org/10.18637/jss.v040.i01

Wickham, H., 2009. ggplot2: Elegant Graphics for Data Analysis, Media. Springer.

Wickham, H., 2007. Reshaping Data with the reshape Package. Journal of Statistical Software, 21(12), 1-20. URL http://www.jstatsoft.org/v21/i12/.

Wickham, H., François, R., Henry, L., Müller, K., 2023. dplyr: A Grammar of Data Manipulation.

Supporting information

S1 Table. List of 307 potential métiers. Includes: main gear (DRB – bivalve dredges, FPO – traps, GNS – gillnets, GTR – trammel nets, LLD – drifting longline, and LLS – bottom longline), scientific name of the target species, number of vessels, number of trips, and number of hauls for the study period (2014-2023).

S1 Fig. Histograms by main gear show the frequency of hauls for each potential métier, in descending order. The main gears represented are: DRB – bivalve dredges, FPO – traps, GNS – gillnets, GTR – trammel nets, LLD – drifting longline, and LLS – bottom longline. Only up to 20 métiers of each gear are represented.

S2 Table. List of the 28 validated métiers. Includes: code for main gear used (DRB – bivalve dredges, FPO – traps, GNS – gillnets, GTR – trammel nets, LLD – drifting longline and LLS – bottom longline), name of the métier (combining the main gear and the target species), scientific name of the target species, number of hauls and levels of the variables that were used to characterize the métier: year (2014-2023), season (spring, summer, autumn and winter), region (northwest, center west, southwest and south), depth (shallow - less than 200 meters, medium - 200–500 meters and deep - more than 500 meters), and sediment type (mud, muddy sand, sandy mud, sand, coarse grained sediment, rock and boulders, mixed sediment and unknown). The values in the body of the table, for the variables used to characterize the métier, correspond to the percentage of hauls in each level of the variable (years 2014-2023). Within each variable, the colors represent the importance of the level (white - [0%-25%[, light green - [25%-50%[, intermediate green - [50%-75%[, and dark green - [75%-100%].

S3 Table. The p-values of the chi-square tests for the goodness-of-fit of the uniform distribution. Includes hauls by year, season, fishing area, depth, and sediment. The Bonferroni correction for multiple tests was applied within each variable.

Fig1

Click here to access/download;Figure;Fig1.tiff

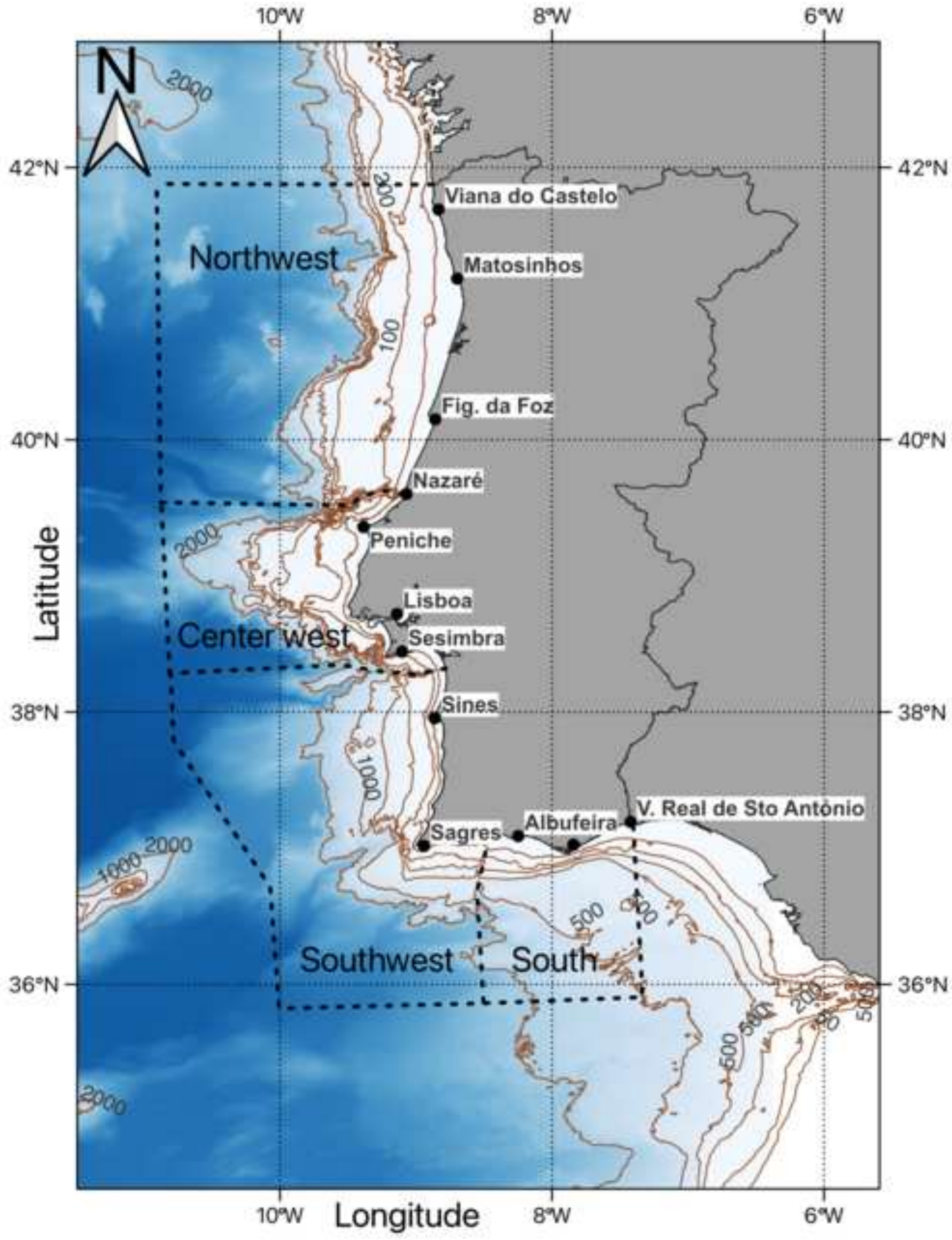

Fig2

Click here to access/download;Figure;Fig2.tiff

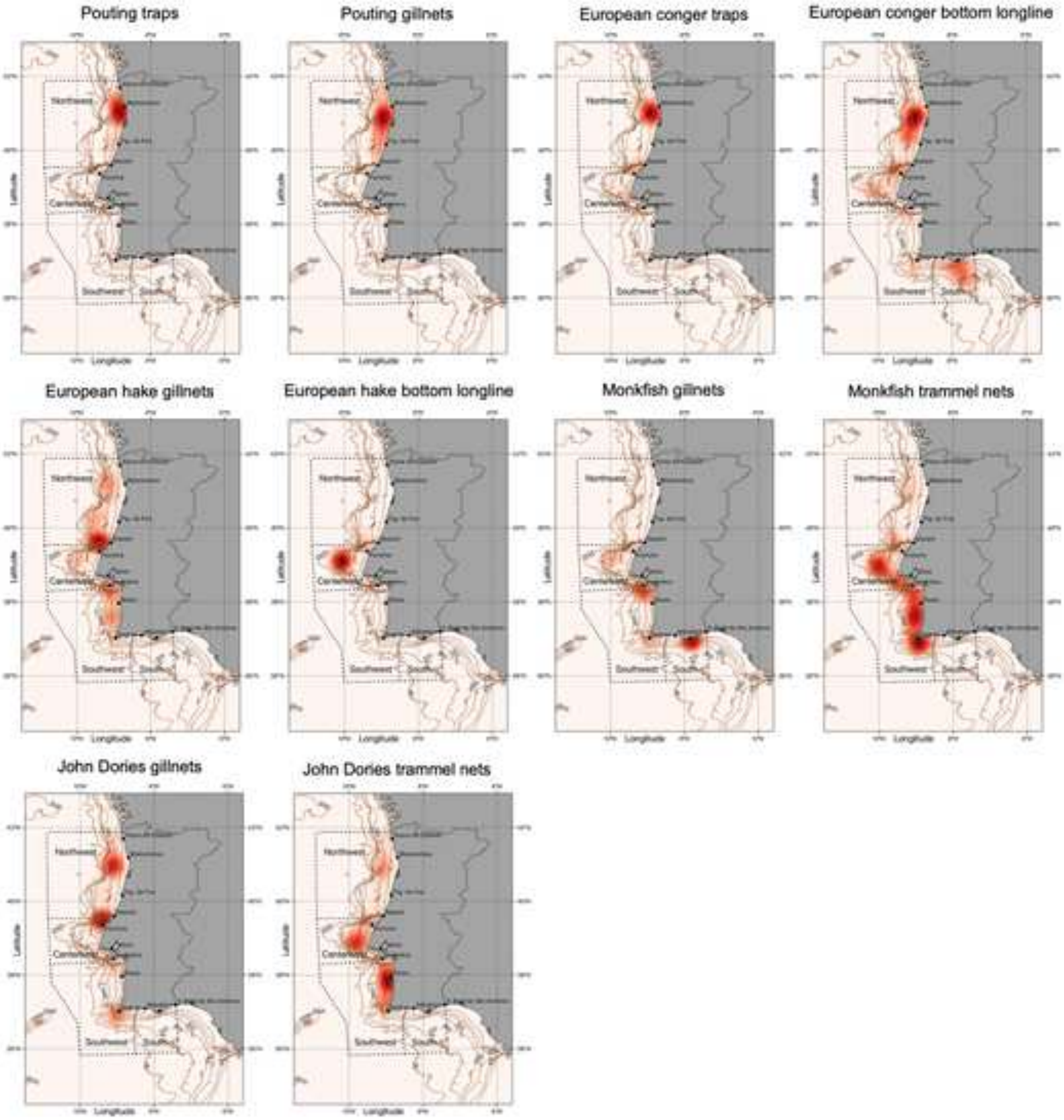

S1 Table

Click here to access/download
Supporting Information
S1 Table.xlsx

S1 Fig

Click here to access/download
Supporting Information
S1 Fig.tiff

S2 Table

Click here to access/download
Supporting Information
S2 Table.xlsx

S3 Table

Click here to access/download
Supporting Information
S3 Table.xlsx
