# Supplementary material for: Identification of métiers in a multi-gear, multi-species fishery
Source: PLoS One. 2026 Jun 2;21(6):e0348392. doi: 10.1371/journal.pone.0348392 (PMC13229354; doi:10.1371/journal.pone.0348392)
Supplement: Attachment — Submitted filename: ReviewersLetter.pdf [file pone.0348392.s008.pdf]

Dear PLOS One reviewers,

Please find attached the reviewed paper and answers to the referees' comments below.

All comments and suggestions were addressed.

Generally, we improved the text as best we could, reading it carefully and running the text through grammar correction tools (DeepL Pro and Grammarly). The most improved section was the statistical analysis, as suggested by the reviewers.

We would like to take this opportunity to thank you for your review work. We appreciate the investment in our paper and recognize that it has substantially improved the work.

Best regards,
Pedro Leitão on behalf of all authors

Reviewer	Line	Comment	Response
2	General	The authors define the target species based on the landed value. However, the authors note that for some of the non-validated metiérs this did not work, because a landed bycatch was much more valuable. Therefore, I wondered how different the validated metiérs would be if the target was defined based on landed weight, and would that have allowed more metiérs to be validated?	Landed value produced the best results, as many species did not show up when landed weight was used. This was the case for gear targeting Nephrops, John Dories or Turbots, which have a high price per kilogramme, are the main target of fishermen and do not have the highest weight. The text was modified at the beginning of section 2.4 to clarify this point.
Unknown	General	In the validation of metiérs, it is currently unclear to me how many skippers were asked per metiér? And, if more than one was asked, what would happen if some says "yes" and some say "no"? Further, it was not clear to me how many of the non-validated metiérs that were due to "no" answers and how many that could not get an answer?	For this work, we used a contact sheet with the names of over 50 skippers and the vessels and gear they operate. When validating the types of gear (e.g. gillnets for hake), we asked skippers who operated that type of gear if they (or their colleagues used it for hake). Different skippers never gave different answers with respect to the same gear. The number of interviews per métier was added to Table 2.
2	General	My understanding is that the new procedure proposed defines a finer scale /lower level metiér than what has previously been used. I think it would be illustrative to compare the new procedure to what was previously done - for example by adding the previous metiér in table 2.	Previously only the main gear was identified. The last paragraph of the introduction was modified to clarify this issue

Unknown	General	The validation of métiers is not clear. The authors mention they used “previous studies, onboard data observations, and interviews with fishers” although they do not explain which type of validation and how was the information used for each métier. Were the answers to the interviews with skippers pooled ? How? What onboard data observations were used and how was the information combined with interviews?	Only one type of validation was used for each métier. The validation options were clarified in the Methods section. Table 2 shows the references of the identified studies.
Unknown	General	Moreover, the approach for the identification of métiers appears to be based on single target species which seems to contradict the multispecies targeting nature of this kind of fisheries and neglect results from previous studies by the same authors. In fact, from the ca. 300 métiers initially identified, the authors end validating 28 métiers suggesting that the initial groups were not true métiers. Although the approach to select métiers is acceptable it is also subjective. I suggest to evaluate the sensitivity of the results to the cutting point to ensure that the results are robust.	This is a valid point, but investigating all 307 potential métiers would be impossible. The cut-off point considered for each main gear. based on the frequency with which each main gear was used. As mentioned in the results, this criterion accounted for 94% of the hauls. The discussion includes important information on this issue. It is correct to assume that the bycatch species sometimes has the highest value. Situations like this would result in an invalidated métier, as mentioned in the discussion.

4	General	The variation of fishing effort with year, season, fishing area, depth, and sediment is not fully supported by a statistical analysis. The authors apply homogeneity tests using variables separately neglecting potential interactions between them (which are likely, for example, between fishing area, depth and sediment type). Moreover, the comparisons between levels of the variables are not statistically tested (statements such as “effort increased over time” are not supported. Authors could explore GLMs to perform the analyses and overcome this gap.	This is a valid point. Initially, we considered using log-linear models for the categorical variables (region, season, depth and sediment type). However, we encountered a problem resulting from multiple categories with very low frequencies (often zero) for levels of the variables and combinations of the levels of different variables. In an attempt to keep the analysis simple, chi-square tests were performed on the main variables, and situations where the test could not be performed (because of low frequencies) are identified in Annex 3. According to the definition of métier, the two most important variables to characterise the métier are region and season. In this case, a test of independence was performed, identifying the situations in which the test could not be performed due to one or more low frequencies. With respect to
3	L013-29	Abstract: Include results in the abstract	The abstract was modified to include main results
Unknown	L045	Sounds a bit out of context of the sentence. Suggest to add a new sentence with this information	The text was modified to consider this comment
Unknown	L049	English doesn't sound good	The sentence rewritten
Unknown	L069	Please clarify the concept of "main commercial species". without a definition it is difficult to evaluate the importance of the fleet.	The sentence was misleading. Data is actually for all landed species and the text was corrected.
Unknown	L072	The authors probably mean Scomber colias, the Atlantic chub mackerel. Please add the scientific names of species the first time (and only the first time) you mention them.	Yes, the species name corrected and the text was revied to take into consideration the comment

Unknown	L076	Does location refer to setting and hauling as well ?	Location refers to the midpoint of a line defined by the position of setting and hauling identified in the onboard diaries. Explained in the second paragraph of section
Unknown	L080-81	in different seasons ?	Seasons are not referred with respect to these species in the
Unknown	L098	Please explain what is level 6	Explanation included in the last paragraph of the introduction
3	L101	Métier should be defined earlier when this term is first used.	The 5th paragraph of the introduction was modified to introduce a first definition of métier
Unknown	L132	What do authors mean by administrative data ?	We considered data provided by the administration but the term was removed.
3	L183	Did you specify which months were included in each season and accurately define all variables?	Season was defined based on day following the exact calendar definition
3	L233-235, L2	Please describe the relevant results found in these tables rather than simply telling us what information is in the tables - that is the purpose of the table caption and does not need to be repeated in the text.	The results were reviewed to take into consideration this comment
Unknown	L245	Were the answers to the interviews with skippers pooled ? How? What onboard data observations were used in validation and how was the information combined with interviews?	The material and methods section was modified to clarify these points. Only one methos of validation was used for each métier, first published work, them onboard observations by members of the research team and lastly validation through port interviews or telephone interviews.

3	L257	Chi-Square tests are sensitive to sample size and multiple tests even with Bonferroni corrections. Large sample sizes often produce highly significant results despite having small effect sizes that are potentially irrelevant. For example, Table S3 has nearly all p-values <0.001 even though some within category differences are much smaller than others. A specific example of this is season for common octopus traps (relatively small differences among seasons) and European sea bass trammel nets (large difference among seasons). Yet, both of these have p < 0.001, which is likely due to the extremely large sample size of hauls for octopus traps, but which also questions the sample size bias in the results. Your current statistical approach treats every haul as independent, which is not true because you have pseudo-replication from repeated measures of the same vessel, year, etc. when assessing differences among season. Did you consider a more robust and flexible modeling approach such as Poisson regression?	This is a valid point. Initially, we considered using log-linear models for the categorical variables (region, season, depth and sediment type). However, we encountered a problem resulting from multiple categories with very low frequencies (often zero) for levels of the variables and combinations of the levels of different variables. In an attempt to keep the analysis simple, chi-square tests were performed on the main variables, and situations where the test could not be performed (because of low frequencies) are identified in Annex 3. According to the definition of métier, the two most important variables to characterise the métier are region and season. In this case, a test of independence was performed, identifying the situations in which the test could not be performed due to one or more low frequencies. With respect to changes over time (years), simple linear regression was used. [illegible]
Unknown	L259	region?	The tern fishing area corresponds to region?
Unknown	L271	the high number of métiers questions the approach to identify potential metiers	This is a valid point, but investigating all 307 potential métiers would be impossible. The cut-off point considered for each main gear. based on the frequency with which each main gear was used. As mentioned in the results, this criterion accounted for 94% of the hauls. The discussion includes important information on this issue. It is correct to assume that
Unknown	L275-278	Please summarise the main patterns instead of describing the table.	The results were reviewed to take into consideration this comment

3	L280	What was the percent accuracy of the métiers that were validated? This is important to inform how accurate the remaining nine were. You do speak to the potential cause of non-validation on L423-424, which is confusing since this topic was not mentioned until then. remaining nine were. You do speak to the potential cause of non-validation on L423-424, which is confusing since this topic was not mentioned until then.	The results and discussion were modified to clarify these points
Unknown	L280-282	please expand on the validation work	The methodology was modified to include a clarification of this point
Unknown	L285-288	Provide the main findings from each table please instead of mainly repeating the Table legend	The results were reviewed to take into consideration this comment
3	L298	What is meant by most representative? Most number of vessels, most valuable, etc.?	Most representative in terms of number of hauls. The text was modified to clarify this point
Unknown	L298	Please clarify what is meant by representative ? The characteristic being used is the number of hauls. It would be interestng to associate the catch in value and the number of vessels to evaluate the importance	Most representative in terms of number of hauls. The text was modified to clarify this point.

3	L300	Here and elsewhere, species or taxa are mentioned without including scientific names or genera/families where appropriate. When you first mention species groups in the body of the manuscript, you should direct the reader to the supplementary tables where the scientific names are included. appropriate. When you first mention species groups in the body of the manuscript, you should direct the reader to the supplementary tables where the scientific names are included.	The scientific names were included when the species was referred for the first time
3	L316 - 336	Throughout these paragraphs, quantitative values should be included within text to support statements of “almost exclusively”, “often”, “strongly associated”, etc. and then parenthetically reference a table where the reader can find more quantitative results. Also, include the depth you are referring to when mentioning “shallow”	The text was modified to accommodate the comment. The depth strata we identified in the legend of Table 2 and in the Methods section
3	L338	Similar to my comment above, please do not start a paragraph or sentence simply stating what information is in a figure or table.	This was corrected
Unknown	L338	why were these species selected ?	This species were selected because they are the only ones target by more than one métier

Unknown	L413-416	Has the exclusion of these métiers been mentioned in M&M ? Please review the paragraph. It is said that these métiers were not analysed in this study but afterwards it is said "our results agree with ...."	These métiers were not excluded. They were the first to be validated because they had been described in published work.
Unknown	L476-478	Please re-phrase and provide more information. What is meant by fixed ? the national sampling program of what ? misendentification at the auctions ended ?	The port authorities were trained to improve the identification od skates but that did not happen in the rest of the country. Text was clarified.
3	L477	Likely replace “fixed the” with “reduced”. It is unlikely that all misidentifications were “fixed” so they no longer occur, correct?	The port authorities were trained to improve the identification od skates but that did not happen in the rest of the country. Text was clarified.
Unknown	L482	This number needs to be mentioned before for clarification.	This information was included in the Methods section 2.3, where the fleet of interest was described
3	L515 – 518	If this is a requirement already in place by the EU, then this concluding statement is not informative, but is instead simply restating a known fact published elsewhere. It would be more far more informative to use results from your study to provide recommendations of which fisheries are most lacking of information and should be prioritized for this effort – or any other recommendations learned from your study that are not already described elsewhere.	To our knowledge, the requirement to all vessels of the coastal fleet having electronic logbooks will only be enforced in 2030. Then the remaining 2/3 of the fleet will have electronic logbooks.

3	L727	There is no description of what the vertical line in each panel refers to	The legend was modified to explain this was the cut point of the métiers considered in the study.
Unknown	Table1	Please add totals to all columns	The total for the number of vessels and trips leads to erroneous information because the same vessel uses more than one gear sometimes in the same trip. An explanation why totals were not included for these
Unknown	Table2	why is there a - in main seaso of most fisheries and main sediment of some.	Sediment is missing for many hauls. Table 2 was reviewed and completed as much as possible.
